# Norepinephrine Administration Is Associated with Higher Mortality in Dialysis Requiring Acute Kidney Injury Patients with Septic Shock

**DOI:** 10.3390/jcm7090274

**Published:** 2018-09-12

**Authors:** Ying-Ying Chen, Vin-Cent Wu, Wei-Chieh Huang, Yu-Chang Yeh, Mai-Szu Wu, Chiu-Ching Huang, Kwan-Dun Wu, Ji-Tseng Fang, Chih-Jen Wu, The NSARF, CAKS Group

**Affiliations:** 1 Division of Nephrology, Department of Internal Medicine, MacKay Memorial Hospital, No. 92, Sec. 2, Zhongshan North Road, Taipei 10449, Taiwan; akochen45@hotmail.com; 2 Graduate Institute of Clinical Medicine, College of Medicine, National Taiwan University, No.7.Chung San South Road, Taipei 10002, Taiwan; 3 Division of Nephrology, Department of Internal Medicine, National Taiwan University Hospital, No.7.Chung San South Road, Taipei 10002, Taiwan; q91421028@ntu.edu.tw (V.-C.W.); kdwu@ntuh.gov.tw (K.-D.W.); 4 Division of Cardiology, Department of Internal Medicine, Taipei Veterans General Hospital, No.201, Sec. 2, Shipai Road, Taipei 11217, Taiwan; hwcc0314@gmail.com; 5 Department of Anesthesiology, National Taiwan University Hospital, No.7.Chung San South Road, Taipei 10002, Taiwan; tonyyeh@ntuh.gov.tw; 6 Division of Nephrology, Department of Internal Medicine, Taipei Medical University-Shuang Ho Hospital, No.291, Zhongzheng Road, Zhonghe District, New Taipei City 23561, Taiwan; maiszuwu@gmail.com; 7 Division of Nephrology, Department of Internal Medicine, China Medical University Hospital, No. 2, Yude Road, North District, Taichung 40447, Taiwan; cch@mail.cmuh.org.tw; 8 Chang Gung University College of Medicine, Taoyuan, Taiwan; Kidney Research Center, Department of Nephrology, Chang Gung Memorial Hospital, 5. Fu-Hsing Street. Kuei Shan Hsiang, Taoyuan City 333, Taiwan; fangjits@cgmh.org.tw; 9 Department of Medicine, Mackay Medical College, No.46, Sec. 3, Zhongzheng Road, Sanzhi District, New Taipei City 252, Taiwan; 10 Graduate Institute of Medical Sciences and Department of Pharmacology, School of Medicine, College of Medicine, Taipei Medical University, 250 Wuxing St., Taipei 11031, Taiwan; 11 Department of Medical Research, China Medical University Hospital, China Medical University, No.91, Hsueh-Shih Road, Taichung 40402, Taiwan; 12 Mackay Junior College of Medicine, Nursing and Management, No.92, Shengjing Road, Taipei 11260, Taiwan

**Keywords:** critical care, vasoactive agents, norepinephrine, sepsis, acute kidney injury, dialysis

## Abstract

(1) Background: Norepinephrine (NE) is the first-line vasoactive agent used in septic shock patients; however, the effect of norepinephrine on dialysis-required septic acute kidney injury (AKI-D) patients is uncertain. (2) Methods: To evaluate the impact of NE on 90-day mortality and renal recovery in septic AKI-D patients, we enrolled patients in intensive care units from 30 hospitals in Taiwan. (3) Results: 372 patients were enrolled and were divided into norepinephrine users and non-users. After adjustment by Inverse probability of treatment weighted (IPTW), there was no significant difference of baseline comorbidities between the two groups. NE users had significantly higher 90-day mortality rate and using NE is a strong predictor of 90-day mortality in the multivariate Cox regression (HR = 1.497, *p* = 0.027) after adjustment. The generalized additive model disclosed norepinephrine alone exerted a dose–dependent effect on 90-day mortality, while other vasoactive agents were not. (4) Conclusion: Using norepinephrine in septic AKI-D patients is associated with higher 90-day mortality and the effect is dose-dependent. Further study to explore the potential mechanism is needed.

## 1. Introduction

Sepsis is defined as a life-threatening organ dysfunction due to dysregulated host response to infection in accordance to recent Sepsis-3 consensus [1]; it is the leading cause of acute kidney injury (AKI) in critically ill patients in intensive care units (ICU) [2,3]. Using sepsis-3 criteria, analysis of data from a large cohort from 409 hospitals in the USA from 2004–2009 revealed that more than 40% of patients with sepsis also had AKI [4]. Septic AKI is associated with high mortality [3,5,6], extended hospitalization [5], and costly long-term treatment [7]. 

Septic shock, which is a condition of systemic vasodilatation and arterial hypotension, is now evidenced by a serum lactate level >2 mmol/L and vasopressor requirement to maintain a mean arterial pressure (MAP) of at least 65 mm Hg in the absence of hypovolemia [4]. The use of vasopressors is necessary, especially when fluid resuscitation fails to restore arterial blood pressure; they are still the cornerstone of hypotension management in patients with septic AKI for maintaining adequate organ perfusion [8,9]. Although norepinephrine (NE) is still recommended as the first-line vasoactive agent used in these patients [10], little is known about the impact of NE and other vasoactive agents on the progression of septic AKI. 

However, in AKI patients with septic shock, the different effects of NE, and other vasoactive agents has not been surveyed. In this study, we compare the impact of NE and other vasoactive agents on mortality and dialysis dependency in acute kidney injury patients who require dialysis (AKI-D).

## 2. Material and Methods

### 2.1. Study Design and Population

The nephrologists and intensivists in Taiwan have appealed for the development of a consortium to unite strength in the field of critical nephrology in Taiwan. The Consortium for Acute Kidney Injury and Renal Diseases (CAKs) and a division focusing on AKI (CAKs-AKI) were launched in the beginning of 2014. This study group has established a multicenter database since 2002 to improve the quality of care and the prognosis of AKI in critically ill patients and then set up a national registry program of AKI to prospectively enroll a large number of dialysis-requiring AKI (AKI-D) patients. This nationwide epidemiology and prognosis of AKI (NEP-AKI-D) requiring dialysis study is the first flagship study of CAKs-AKI, which aims to explore the epidemiology, risk factors, modality, dose, and frequency of renal replacement therapy (RRT), as well as prognoses of the patients with AKI-D, by using the established anonymous nationwide AKI database launched in the beginning of 2014. Up to January of 2016, 30 hospitals have joined this consortium. These hospitals are distributed widely through the four geographical regions (north, middle, south, and east) of Taiwan, and have a 1:1 ratio of medical centers to regional hospitals in each region [11]. In the included hospitals, adult patients fulfilling the diagnosis of septic shock according to the sepsis-3 criteria [1] at initialized RRT in the ICU were prospectively enrolled in the study and followed until hospital discharge from the six seasonally sampled months (October 2014, along with January, April, July, October 2015, and January 2016). Patients who had ever received dialysis treatment or arteriovenous creation before the index hospitalization were excluded. The use of vasoactive agents was assessed, and septic shock patients were separated into NE (NE) users and other inotropic users (NE nonusers). The outcomes of interest were 90-day mortality and recovery from dialysis-dependency after hospital discharge. 

### 2.2. Data Collection and Variable Definitions

Sepsis is defined in Sepsis-3 as life-threatening organ dysfunction, which is known as an acute change in total SOFA score ≥2 points, caused by a dysregulated host response to infection. And septic shock is defined by a serum lactate level >2 mmol/L and persistent hypotension after fluid resuscitation; it also requires vasopressors to maintain MAP >65 mmHg [1,4].

Other organ failure is classified as the following [12,13]: (1) respiratory failure: requiring ventilator support; (2) central nervous system failure: Glasgow Coma Score <9; (3) cardiac failure: signs of low cardiac output with a central venous pressure >12 mmHg; and (4) liver dysfunction: total bilirubin >2.0 mg/dL and international normalized ratio (INR) >1.4 [12]. 

Disease severity was assessed by using the Acute Physiology and Chronic Health Evaluation II (APACHE II) score [14], the Sequential Organ Failure Assessment (SOFA) score [15], and inotropic equivalent (IE) score [16]. We also recorded ICU procedure, infection site, the use of vasoactive agents, and laboratory data at the time of dialysis initiation. We defined “baseline serum creatinine (SCr)” as the latest SCr value during outpatient department (OPD) follow-up for patients who had not visited an OPD within 6 months before index admission. The etiologies of AKI, which included sepsis and other etiologies in the meantime, were documented as well. RRT in this study was performed via a double-lumen catheter. The modality of RRT was chosen according to clinical judgment of the consulting nephrologist and the in-charge intensivist. (Supplemental methods) Because the type and dosage of catecholamines preferred by physicians can vary, this study compared the dosage of catecholamines according to inotropic equivalents (IE, µg/kg/min = dopamine + dobutamine + 100 × epinephrine + 100 × NE + 100 × isoproterenol + 15 × milrinone) in order to compare the severity of heart failure, [16] which was composed of most common used vasoactive agents nowadays. This score had been used in other studies [16,17] for evaluating the severity of cardiovascular dysfunction and it is a valid surrogate outcome measure in pediatric sepsis by testing its association with important short-term outcomes [17,18,19,20]. In this study, the vasoactive agents we recorded included dopamine, dobutamine, norepinephrine, epinephrine, isoproterenol, and milrinone. The “other vasoactive agents” were identified as vasoactive agents, except norepinephrine, which was included in the IE score. We evaluated the disease severity before dialysis.

### 2.3. Statistical Analyses

Continuous variables between groups were compared using the Student t test. The chi-square test was applied for categorical variables with Yates’ correction where applicable. The inverse probability of treatment weighting (IPTW) using the propensity score was applied to correct the bias of the two groups in basic characteristics and outcomes [21]. Applying these weights has the effect of creating a pseudo-population with a covariate distribution of the individual treatment groups similar to that of the overall study population. Covariate balance was assessed by examining the magnitude of any residual differences between the treatment groups after applying the weight [22]. Accumulated hazard ratio was modeled by Cox regression models and adjusted for the covariates for the outcomes of interest (Table 1). The significance levels for entry (SLE) and for stay (SLS) were set to 0.15 for being conservative. Then, with the aid of substantive knowledge, the best candidate final logistic regression model was identified manually by dropping the covariates with *p* value > 0.05 one at a time until all regression coefficients were significantly different from 0 [13,23].

Forest plot was constructed for odds ratio of NE use (vs. other vasoactive agents) on 90-day mortality according to prior comorbidities and clinical conditions. The generalized additive model was used to analyze the dose-response relationship between vasoactive agents and the 90-day mortality [24,25]. Because of the high mortality rate in sepsis patients after AKI-D, competing risk regression was also performed to show the risks for dialysis dependence using the Fine and Gray model considering the subdistribution hazard [26,27].

We used R software version 3.2.2 (Free Software Foundation, Inc., Vienna, Austria) for the time-varying Cox model and Stata/MP version 14 (Stata Corporation, College Station, TX, USA) for the competing risk analysis. Two-sided *p* values < 0.05 were considered statistically significant.

### 2.4. Ethics Approval and Consent to Participate

Approval of this prospective multi-center study follows the regulations of the National Research Program for Biopharmaceuticals (NRPB)-Institutional Review Board (IRB). All clinical trial consortiums have to fill in the application forms on the official website of the NRPB-IRB. Written t informed consent was obtained from all participants before inclusion. (Approval No. NRPB2014050014).

## 3. Results

### 3.1. Baseline Characteristics of the Study Cohort

A total of 372 AKI-D patients fulfilled the criteria of septic shock at initialization of dialysis and 315 patients were NE users. The characteristics of patients are listed in Table 1. The mean SOFA score was 15.42 ± 2.69 in the non-user group and 15.28 ± 2.99 in the user group. The APACHE II score was 26.51 ± 6.74 in the user group and 27.21 ± 6.49 in the non-user group. The mean age was 65.8 ± 15.2 years in the non-NE user group and 64.8 ± 15.7 years in the NE user group. After adjustment by IPTW (Table 1), there was no significant difference of baseline comorbidities between the two groups. The biochemical data and ICU procedure were similar. There was the sum of 263 (70.7%) patients who received surgery during admission. Regarding disease severity, there was no significant difference in APACHEII score (25.79 ± 7.57 in NE non-user and 27.27 ± 6.46 in NE user, *p* = 0.289) between the two groups initially, and other scoring systems including IE score and SOFA (15.42 ± 2.69 in NE non-user and 15.28 ± 2.99 in NE user, *p* = 0.663) were similar after adjustment. We record the etiology of acute kidney injury included with/without cardiorenal syndrome, drug, rhabdomyolysis, pigmentation (pigment nephropathy), hepatorenal syndrome, and contrast. And there was no significant difference in the etiologies of AKI between two groups. Besides, no significant difference was noted in major infection site which included respiratory tract, genitourinary tract, blood stream, or abdomen between NE user and non-user. Oliguria (81.9%) is the leading cause for RRT, followed by fluid overloaded (67.8%). The indications for RRT were similar except for oliguria (*p* = 0.009). Regarding the RRT modality, NE users tended to receive CVVH, and SLEDD (*p* = 0.034). The detail of these baseline characteristics is in the Supplementary Table S1.

### 3.2. 90-Day Mortality in Septic-Shock-Related AKI-D

A total of 302 (81.2%) patients died within 90 days of hospital discharge (Table 1) and 260 (92.54%) patients were NE users and 42 (73.68%) were NE non-users. The median ICU stay for NE users was similar to that of NE non-users (*p* = 0.204). The number of dialysis days of the NE non-user groups was larger than that of the NE user group (user vs. non-user = 12.9 ± 19.5 vs. 18.7 ± 27.8 days, *p* = 0.012).

Table 2 showed the independent risk predictors for 90-day mortality analyzed by multivariate Cox proportional hazards model incorporated with IPTW. After adjustment by age, sex, comorbidities, kidney function, APACHE II score, indication for dialysis, and dialysis modalities, the use of NE was an independent risk for 90-day mortality (hazard ratio = 1.504, *p* = 0.026).

**Figure 1:**
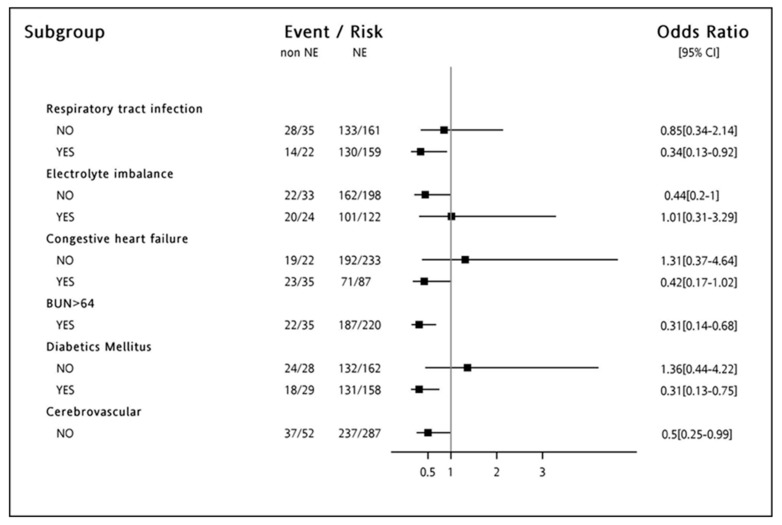
**Odds ratio of Norepinephrine user on the development of 90 days mortality.** Odds ratio of Norepinephrine users (vs. other vasoactive agents) on the development of 90-day mortality, according to demographics in septic shock patients at dialysis initiation. Patients those who had respiratory tract infection, congestive heart failure, diabetes mellitus, BUN with more than 64 mg/dL, and did not have cerebrovascular disease tended to have a higher 90-day mortality while norepinephrine (NE) was prescribed.

We further analyzed the outcomes of the use of NE; subgroup analysis using forest plots was performed to calculate IPTW adjusted HRs (HRs) of 90-day mortality. The detrimental effects of NE were consistent across the subgroups stratified by respiratory tract infection, congestive heart failure, diabetes mellitus, BUN with more than 64 mg/dL, and patients who did not have cerebrovascular disease (Figure 1).

### 3.3. The Dose-Response Relationship between NE and 90-Day Mortality

**Figure 2:**
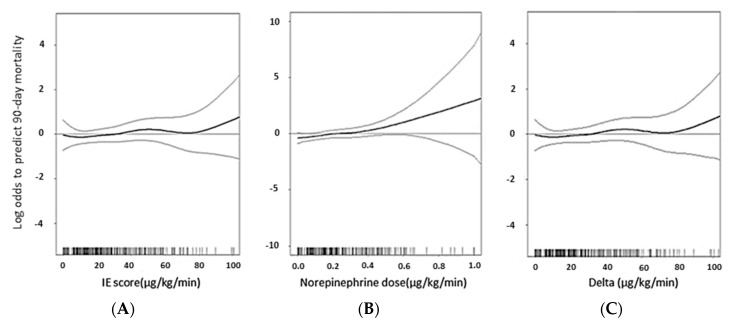
Generalized additive mode (GAM) plot of the probability of 90-day mortality regarding the dose equivalent of inotropes. The model incorporates subject-specific (longitudinal) random effects, expressed as the logarithm of the odd (logit). The probability of mortality was constructed with the equivalent dose of (**A**) inotropes (**B**) NE (**C**) inotropic dose deduct NE dose and was centered to have an average of zero over the range of the data as constructed with the GAM. IE: inotropic equivalent.

The quantity of NE was plotted against the log odds of predicting 90-day mortality by using generalized additive mode (GAM) after adjusting the risk factors in the final model (Figure 2). We separated the use of vasoactive agents into 3 groups by measurement of IE score which included vasoactive agents mentioned above (Figure 2A), NE alone (Figure 2B), and vasoactive agents other than NE (Figure 2C). The dose of total vasoactive agents and those without NE did not show a dose-dependent effect relating to 90 day-mortality. However, NE equivalence showed a positive relationship to 90-day mortality. In terms of the risks of 90-day mortality, the GAM plot disclosed with NE alone exerted a significant disadvantage when compared to other vasoactive agents; it was dose-dependent (Figure 2). 

### 3.4. Dialysis Dependency in Septic Shock Patients with or without NE

**Figure 3:**
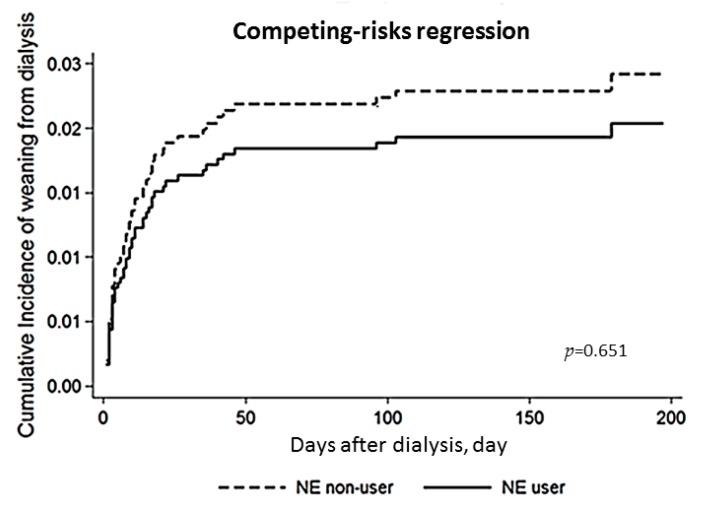
**Cox proportional plot depict cumulative proportions of renal recovery between NE user and non-user groups.** There was no significant difference (*p* = 0.651) in weaning from dialysis between NE user group and non-user group when taking mortality as a competing risk. BUN: blood urea nitrogen; GAM: generalized additive model; NE: norepinephrine.

In the evaluation of the relation between the use of NE and dialysis dependency, we conducted a competing-risk regression model which took mortality as a competing risk (Figure 3). It revealed that the use of NE was not associated with the cumulative proportions of weaning from dialysis (*p* = 0.651).

## 4. Discussion

### 4.1. Main Finding

In this multi-center, observational study of AKI-D patients with septic shock at initialization of dialysis, we found that 81.2% of patients died within 90 days of hospital discharge. Our report first showed the high mortality of AKI-D patients with septic shock according to Sepsis-3 criteria. The use of NE was associated with a higher 90-day mortality rate than other vasoactive agents and that the detrimental effect is dose-dependence. We applied inverse probability of treatment weighting (IPTW), to minimize the effect of confounding in observational study [28]. And importantly, the risk of NE on 90-day mortality is constant after adjusting for the bias of baseline characteristics by using inverse probability of treatment weighting.

### 4.2. The Use of NE in AKI-D Patients with Septic Shock

In the general population, the use of NE is the first-line choice of vasoactive agents to restore organ perfusion and maintain the blood pressure in septic shock patients [29]. The influence of the use of NE on AKI-D patients with septic shock is still warranted.

Chou et al [30]. conducted a retrospective study which prescribed a high-dose of vasopressor used before the initiation of continuous renal replacement therapy (CRRT). A high dose of NE is associated with higher mortality by way of the catecholamine effect on the cardiovascular system [31]. In a multi-center, double-blind, randomized, controlled trial, vasopressin reduce progression to mortality in early stage AKI patients when compared to NE [32].

Thus, differences between vasopressin and nor epinephrine-treated patient outcomes may be due to the beneficial effects of vasopressin or, alternatively, due to reduction in the detrimental effects of norepinephrine. This result is also consistent with the primary subgroup analysis of the VASST study in which vasopressin treatment was associated with decreased mortality in patients who had less severe shock and not in patients who had more severe shock [33].

In a recent prospective cohort study [34], Passos et al. tried to establish a scoring system to predict 7-day mortality in septic patients requiring CRRT. The use of norepinephrine was recognized as one of the predictors in the study based on the magnitude of regression coefficients in the multivariate analysis. One multi-center, prospective, observational study which enrolled 897 patients with community-acquiring sepsis from seventeen Portuguese ICUs evaluated the impact of vasopressor on mortality. The study reported that the use of NE, either used as single agents or in combination, was associated with worse outcome due to increased cardiovascular events when compared to dopamine in community-acquired sepsis patients during ICU stay [35].

Adequate fluid resuscitation is recommended and it is closely associated with blood pressure maintenance with the ultimate aim of maintaining tissue perfusion and oxygenation [36]. However, insufficiency of fluid resuscitation insufficiency may occur in oliguric septic AKI-D patients because clinician are often anxious to aggressive hydration related fluid overload. One retrospective study enrolled dialysis patients who had sepsis revealed severely under resuscitated and it might contribute to the patients’ mortality [37]. To maintain the hemodynamic status, even in dialysis patients, clinicians should add sufficient fluid supplement before adding more vasoactive agents [37]. In addition to fluid resuscitation, antimicrobials therapy should be initiated as soon as possible when the diagnosis of sepsis and septic shock is established [10].

In our study we found worse outcomes when using NE in AKI-D patients with septic shock when compared to other vasoactive agents, even after adjustment for the disease severity. Besides, the disease severity was more severe in our study population in accordance with SOFA score and APACHEII score which might lead to higher mortality rate in our study. In previous study, the highest SOFA scores, higher than 11, were associated with a mortality rate greater than 80% [38]. The mean SOFA score was 15.6–2.96 in the NE user group and 14.02–2.66 in the NE non-user group in our study. Besides, almost 80% patients were from a medical center that assembled very ill patients in our country. Septic shock patients who were not candidates for NE could differ from patients who were candidates, and it will be noted as an indication bias in this observational study. However, the disease severity score, the dose of inotropic equivalent, and even the level of serum lactate, in terms of the severity of septic shock, were similar between the two groups. Therefore, the indication bias, if any, will be trivial in this observational study. NE was known as a more potent vasopressor than dopamine and had favorable outcome on mortality in earlier studies [39]. However, one current large randomized trial—the SOAP II trial, demonstrated that there was no significant difference-in 28-day mortality between the use of NE and dopamine in patients with shock. The result was similar in septic shock and hypovolemic shock, except in cardiogenic shock when the researchers conducted subgroup analysis [40]. Recently, the NE as Initial Therapy in Septic Shock (VANISH) trial [41] reported that AKI occurred in about 45% of patients, and AKI requiring RRT developed in 30% of patients. Although the clinical use of inotropic agents is common in patients with septic AKI requiring dialysis, little was known about which inotropic agent is preferable in these patients. In light of our study, the use of NE in septic AKI requiring dialysis was associated with higher 90-day mortality, and the detrimental effect was dose-dependent. Further study is warranted to reconsider the early use of NE in AKI-D patients with septic shock.

### 4.3. NE and Renal Recovery

In general, the infusion of NE deceases the renal blood flow [42] and renal vasoconstriction, which may lead to reversible AKI [43]. In light of our study, the vasopressin and septic shock trial (VASST) did not show any difference in the incidence of AKI or need for RRT with the use of vasopressin or NE [33].

However, in acute endotoxemic status, the infusion of NE appears to improve renal blood flow, and had favorable effects on renal function in septic patients. One recent animal study [44] tried to evaluate the NE effects on the kidney in septic AKI ovine, and it found that medullary hypoxia and ischemia were exacerbated after NE infusion. In our study, we showed that the use of NE could not be the crucial factor for recovery from dialysis.

### 4.4. Study Limitation

This is the first study evaluating NE in critical AKI-D patients with septic shock according to the sepsis-3 criteria from a nationwide cohort. We followed up patients with septic AKI on dialysis to 90 days after hospital discharge, and further evaluated the recovery from AKI promptly. However, our study still had some limitations. First, our study did not evaluate the catecholamine-sparing effect of NE, in addition to the different patient population; and we did not evaluate the vascular response to NE under critical dialysis. Secondly, the indication for the use of NE or other vasoactive agents is not standardized. However, due to the difficulty in the randomness of enrolling patients with septic shock, an observational study could provide valuable information. Third, there could be a selection bias in choosing vasoactive agents due to current guidelines showed that norepinephrine is the first choice for septic shock and low dose dopamine for prophylactic use is no longer recommended. Last but not the least, we did not record pre-dialysis data of hemodynamic status, such as central venous pressure or volume of fluid resuscitation completely. Therefore, we could not evaluate the impact of fluid status when dialysis-required septic AKI developed and the association with the vasoactive agent usage to mortality.

## 5. Conclusions

The use of NE in septic AKI patients at the initialization of dialysis is associated with a higher 90-day mortality after we adjusted for severity by IPTW compared to other vasoactive agents, and the detrimental effect was dose-dependent. Therefore, consideration of early treatment to block septic AKI vicious cycle to stabilize the hemodynamic status should nonetheless precede increased doses of norepinephrine at dialysis initiation. Further study to explore the potential and possible mechanism is needed.

## Figures and Tables

**Table 1:** Comparison of baseline characteristic and outcomes of septic shock patients with norepinephrine (NE) or other vasoactive agents at dialysis initiation.

Variables	Before IPTW			After IPTW		
	NE Non-User (*n* = 57)	NE User (*n* = 315)	*p* * Value	NE Non-User (*n* = 57)	NE User (*n* = 315)	*p* * Value
**Age (year)**	65.79 ± 15.22	64.77 ± 15.72	0.637	66.56 ± 14.53	64.66 ± 15.95	0.587
**Gender (male)**	36 (63.16%)	224 (71.11%)	0.272	21 (31.11%)	91 (29.82%)	0.797
**DM**	29 (50.88%)	155 (49.21%)	0.886	29 (47.73%)	155 (50.00%)	0.772
**CAD**	17 (29.82%)	65 (20.63%)	0.163	17 (22.22%)	65 (22.59%)	0.946
**CVA**	5 (8.77%)	33 (10.48%)	0.816	5 (11.11%)	33 (16.27%)	0.293
**CHF**						
**I**	13 (22.81%)	3 (5.26%)	<0.001	13 (31.11%)	3 (41.99%)	0.551
**II**	12 (21.05%)	153 (48.57%)		12 (28.89%)	153 (22.66%)	
**III**	12 (21.05%)	68 (21.59%)		12 (26.67%)	68 (16.92%)	
**IV**	17 (29.82%)	59 (18.73%)		17 (11.11%)	59 (10.57%)	
**BUN (mg/dL)**	57.59 ± 47.42	50.19 ± 40.14	0.225	51.01 ± 49.62	50.19 ± 39.48	0.700
**Lactate(mmol/L)**	10.65 ± 9.03	8.77 ± 6.98	0.160	9.14 ± 8	9.62 ± 7.36	0.734
**Baseline Cr (mg/dL)**	1.62 ± 1.16	1.36 ± 1.02	0.074	1.65 ± 1.18	1.37 ± 1.03	0.254
**eGFR (mL/min/1.73 m^2^)**	57.03 ± 35.28	69.63 ± 46.76	0.048	57.34 ± 36.15	68.12 ± 44.9	0.319
**Etiology of AKI**						
**Shock**	29 (50.88%)	242 (76.83%)	<0.001	29 (60.00%)	242 (68.37%)	0.306
**CRS**	34 (59.65%)	82 (26.03%)	<0.001	34 (35.56%)	82 (33.13%)	0.663
**Drug**	2 (3.51%)	21 (6.67%)	0.551	2 (8.89%)	21 (5.72%)	0.363
**Rhabdomyolysis**	4 (7.02%)	43 (13.65%)	0.198	4 (4.44%)	43 (13.86%)	0.054
**Pigmentation**	0 (0.00%)	14 (4.44%)	0.140	0 (0.00%)	14 (6.02%)	0.092
**Hepatorenal**	4 (7.02%)	23 (7.30%)	0.999	4 (8.89%)	23 (6.93%)	0.648
**Contrast**	4 (7.02%)	24 (7.62%)	0.999	4 (11.11%)	24 (10.54%)	0.956
**Others**	4 (7.02%)	33 (10.48%)	0.630	4 (4.44%)	33 (9.04%)	0.299
**Infection site**						
**Respiratory**	22 (38.60%)	157 (49.84%)	0.149	22 (46.67%)	157 (46.08%)	0.999
**GU**	19 (33.33%)	77 (24.44%)	0.188	19 (20.00%)	77 (22.36%)	0.738
**Bacteremia**	12 (21.05%)	96 (30.48%)	0.204	12 (26.67%)	96 (28.61%)	0.903
**Abdomen**	3 (5.26%)	53 (16.83%)	0.026	3 (6.67%)	53 (14.16%)	0.157
**Others**	6 (10.53%)	39 (12.38%)	0.827	6 (8.89%)	39 (12.95%)	0.435
**Disease severity score**						
**Total IE Score**	13.97 ± 14.11	31.99 ± 25.36	<0.001	24.23 ± 23.62	29.17 ± 24.21	0.261
**SIRS**	2.7 ± 0.89	2.92 ± 0.83	0.080	2.87 ± 0.95	2.87 ± 0.81	0.822
**SOFA**	14.02 ± 2.66	15.6 ± 2.96	<0.001	15.42 ± 2.69	15.28 ± 2.99	0.663
**qSOFA**	2.3 ± 0.46	2.37 ± 0.48	0.311	2.25 ± 0.45	2.35 ± 0.48	0.300
**APACHEII**	25.79 ± 7.57	27.27 ± 6.46	0.289	26.51 ± 6.74	27.21 ± 6.49	0.851
**Outcome**						
**ICU day**	28.65 ± 38.07	17.22 ± 17.88	0.014	30.94 ± 37.27	17.58 ± 18.32	0.204
**Length of hospital dialysis**	26.07 ± 36.97	12.68 ± 19.19	<0.001	18.67 ± 27.78	12.85 ± 19.47	0.012
**Hospital Mortality**	41 (71.93%)	252 (80.00%)	0.217	41 (75.56%)	252 (81.02%)	0.330
**90-day outcome**			0.142			0.753
**Mortality**	42 (73.68%)	260 (82.54%)		42 (76.09%)	260 (83.13%)	
**Recovery from dialysis**	13 (22.81%)	52 (16.51%)		13 (21.74%)	52 (15.66%)	
**Dialysis-dependent**	2 (3.51%)	3 (0.95%)		2 (2.17%)	3 (1.20%)	

Abbreviations: AKI: acute kidney injury; APACHEII: acute physiology and chronic health evaluation; BUN: blood urea nitrogen; CAD: coronary artery disease; CHF: congestive heart failure; Cr: creatinine; CRS: cardiorenal syndrome; CVA: cerebrovascular accident; DM: diabetes mellitus; eGFR: estimated glomerular filtration rate; GU: genitourinary; IABP: intra-aortic balloon pump; ICU: intensive care unit; IE: inotropic equivalent; IHD: intermittent dialysis; IPTW: inverse probability of treatment weighting; qSOFA: quick sequential organ failure assessment; SOFA: sequential organ failure assessment; SIRS: systemic inflammatory response syndrome. * All statistics were two-tailed, and significance was accepted for *p* < 0.05.

**Table 2:** Cox regression for 90-day m4.ortality in septic shock patients with NE or other vasoactive agents at dialysis initiation *.

Variables	HR	Lower 95% CI	95% CI	*p*
NE user (yes)	1.497	1.046	2.141	0.027
Hepatorenal syndrome (yes)	1.992	1.320	3.007	0.001
Cr (mg/dL)	0.840	0.776	0.910	<0.001
BUN (mg/dL)	1.008	1.004	1.012	<0.001
APACHEII Score	1.042	1.030	1.836	<0.001

Concordance = 0.645; R square = 0.152; APACHEII: acute physiology and chronic health evaluation; BUN: blood urea nitrogen; CHF: congestive heart failure; CI: confidence interval; Cr.: creatinine; HR: hazard ratio; NE: norepinephrine; NYHA: New York heart association; * Cox proportional hazard model adjusted with IPTW.

## Supplementary Materials

The following are available online at http://www.mdpi.com/2077-0383/7/9/274/s1.
