# Supplementary material for: Norepinephrine Administration Is Associated with Higher Mortality in Dialysis Requiring Acute Kidney Injury Patients with Septic Shock
Source: J Clin Med. 2018 Sep 12;7(9):274. doi: 10.3390/jcm7090274 (PMC6162856; doi:10.3390/jcm7090274)
Supplement: Supplementary file 1 [file jcm-07-00274-s001.zip › Supplementary file.docx]

**Supplementary file**

**Supplementary file 1:**

**Supplementary Material and methods:**

Clinical Data Collection

Comorbidities were defined as followed: diabetes mellitus (DM): previous usage of insulin or oral hypoglycemic agents; heart failure: New York Heart Association functional class IV; chronic kidney disease (CKD): baseline estimated glomerular filtration rate (eGFR) ≦ 60 mL/min/1.73m2 for more than 3 months

Definition of basal kidney function

We defined baseline serum creatinine (sCr) as the value obtained at hospital discharge at the previous admission in those who had more than one admission, or the value estimated using the Modification of Diet in Renal Disease equation in those without previous records (assuming an average eGFR of 75 ml/min/1.73m2) (1). The peak sCr was defined as the highest sCr before RRT initiation in ICUs.

Dialysis setting

The RRT modalities for individual patients were chosen by the clinical team and occasionally changed later according to patients’ hemodynamics. For those who needed cardiovascular support with catecholamines of more than 15 mcg/kg/min to maintain systemic blood pressure up to 120 mmHg, continuous veno-venous hemofiltration (CVVH) was prescribed. The hemofiltration flow and blood flow were 25ml/kg/hour and 200ml/min, respectively. Replacement fluid was bicarbonate buffer and was pre-dilutionally administered at a dynamically adjusted rate to achieve the desired fluid therapy goals. For patients who required IE of 5-15 mcg/kg/min, sustained low efficiency daily dialysis (SLEDD) or diafiltration (SLEDD-f) was used with blood flow of 200ml/min, dialysate flow of 300ml/min, and hemofiltration flow of 25ml/kg/hour. The duration was around 6-12 hours, depending on the amount of ultrafiltration required. Intermittent hemodialysis was performed for 4 hours except for the first and second sessions. We used low-flux polysulfone hemofilters (KF-18C, Kawasumi Laboratories, Japan) with a dialysate and blood flow of 500ml/min. (2-5) Double lumen catheters were placed for vascular access.

**Table S1.** Comparison of baseline characteristic and of septic shock patients with NE or other vasoactive agents at dialysis initiation

| **Variables** | **Before IPTW** | | | **After IPTW** | | |
| --- | --- | --- | --- | --- | --- | --- |
|  | **NE non-user** | **NE user** | ***P** value** | **NE non-user** | **NE user** | ***P** value** |
|  | (n=57) | (n=315) |  | (n=57) | (n=315) |  |
| **Hospital Level** | | | | | | |
| **Metropolitan** | 8(14.04%) | 67(21.27%) | 0.281 | 8(14.10%) | 67(20.20%) | 0.273 |
| **Medical Center** | 49(85.96%) | 248(78.73%) |  | 49(85.90%) | 248(79.80%) |  |
| ***ICU procedure*** | | | | | | |
| **Surgery (yes)** | 29(50.88%) | 234(74.29%) | 0.001 | 29(51.11%) | 234(66.27%) | 0.053 |
| **Intubation** | 51(89.47%) | 306(97.14%) | 0.016 | 51(95.56%) | 306(94.88%) | 0.903 |
| **IABP** | 9(15.79%) | 23(7.30%) | 0.067 | 9(15.56%) | 23(13.25%) | 0.592 |
| **CPR** | 15(26.32%) | 63(20.00%) | 0.291 | 15(13.33%) | 63(21.39%) | 0.203 |
| **Infection site** | | | | | | |
| **Respiratory** | 22(38.60%) | 157(49.84%) | 0.149 | 22(46.67%) | 157(46.08%) | 0.999 |
| **GU** | 19(33.33%) | 77(24.44%) | 0.188 | 19(20.00%) | 77(22.36%) | 0.738 |
| **Bacteremia** | 12(21.05%) | 96(30.48%) | 0.204 | 12(26.67%) | 96(28.61%) | 0.903 |
| **Abdomen** | 3(5.26%) | 53(16.83%) | 0.026 | 3(6.67%) | 53(14.16%) | 0.157 |
| **Others** | 6(10.53%) | 39(12.38%) | 0.827 | 6(8.89%) | 39(12.95%) | 0.435 |
| ***Indication for dialysis*** |  |  |  |  |  |  |
| **Azotemia** | 17(29.82%) | 112(35.56%) | 0.452 | 17(24.44%) | 112(33.43%) | 0.282 |
| **Fluid overload** | 42(73.68%) | 214(67.94%) | 0.440 | 42(75.56%) | 214(64.46%) | 0.178 |
| **Electrolyte imbalance** | 24(42.11%) | 120(38.10%) | 0.559 | 24(46.67%) | 120(42.77%) | 0.658 |
| **Metabolic acidosis** | 34(59.65%) | 204(64.76%) | 0.458 | 34(62.22%) | 204(65.06%) | 0.773 |
| **Oliguria** | 44(77.19%) | 259(82.22%) | 0.359 | 44(66.67%) | 259(83.43%) | 0.009 |
| **Uremic encephalopathy** | 1(1.75%) | 6(1.90%) | 0.999 | 1(0.00%) | 6(1.81%) | 0.659 |
| ***Dialysis modality*** |  |  | 0.127 |  |  | 0.034 |
| **CVVH** | 37(64.91%) | 227(72.06%) |  | 37(66.67%) | 227(72.89%) |  |
| **IHD** | 17(29.82%) | 59(18.73%) |  | 17(31.11%) | 59(18.98%) |  |
| **SLEDD** | 3(5.26%) | 29(9.21%) |  | 3(2.22%) | 29(8.13%) |  |
